# Interstitial Keratitis, Vertigo, and Vasculitis: Typical Cogan's Syndrome

**DOI:** 10.1155/2014/830831

**Published:** 2014-03-04

**Authors:** Ahad Azami, Nasrollah Maleki, Mohammadreza Kalantar Hormozi, Zahra Tavosi

**Affiliations:** ^1^Department of Internal Medicine, Imam Khomeini Hospital, Ardabil University of Medical Sciences, Ardabil, Iran; ^2^Department of Endocrine and Metabolic Diseases, The Persian Gulf Tropical Medicine Research Center, Bushehr University of Medical Sciences, Bushehr 7514763448, Iran; ^3^Department of Internal Medicine, Shohadaye Khalije Fars Hospital, Bushehr University of Medical Sciences, Bushehr, Iran

## Abstract

Cogan's syndrome (CS) is a chronic inflammatory disorder of unknown etiology that most commonly affects young adults. Clinical hallmarks are bilateral interstitial keratitis and vestibuloauditory dysfunction. Association between CS and systemic vasculitis as well as aortitis also exists. The diagnosis of CS is based upon presence of characteristic inflammatory eye disease and vestibuloauditory dysfunction. We describe classic Cogan's syndrome in a 47-year-old female from Ardabil. The patient was admitted with headache, vertigo, nausea, vomiting, right leg claudication, musculoskeletal pains, bilateral hearing loss, and blindness for the past two months. Ophthalmologic examination revealed that visual acuity was 0.1 bilaterally. Conjunctival hyperemia, bilateral cataract, and interstitial keratitis were detected with a slit lamp examination. Pure tone audiogram (PTA) and auditory brain stem response (ABR) showed bilateral sensorineural hearing loss. The other differential diagnosis of CS was studied and ruled out. Pulse i.v. methylprednisolone and cyclophosphamide were given and were followed by oral prednisolone and cyclophosphamide. Clinical follow-up showed partial improvement.

## 1. Introduction

Cogan's syndrome (CS) is a rare chronic inflammatory disorder characterized by nonsyphilitic interstitial keratitis and vestibuloauditory dysfunction [1]. Associations between CS and systemic vasculitis as well as aortitis also exist [2–4]. The peak incidence of CS occurs in the third decade of life. In the two largest series to date, the median age of onset was 22 years (range 5 to 63 years) [2, 3]. CS may also occur in children and the elderly [5–7]. There is no known gender or racial predominance. Fewer than five percent of patients initially present with systemic manifestations. In these cases, the diagnosis of CS can only be established after the development of eye or inner ear disease [8]. The predominant ocular feature of CS is interstitial keratitis (IK), which typically causes eye redness, pain, photophobia, and blurred vision. On examination of patients with IK, an irregular, granular corneal infiltration is observed, affecting particularly the posterior part of the cornea, near the limbus. Although IK is the classic eye finding, it is not essential for the diagnosis. Ocular inflammation may involve other parts of the eye and lead to iridocyclitis, conjunctivitis, episcleritis, anterior or posterior scleritis, or retinal vasculitis [3, 8–10]. The inner ear manifestations of CS are Ménière's-like attacks consisting of vertigo, ataxia, nausea, vomiting, tinnitus, and hearing loss [11]. Most patients do not develop features of more widespread systemic vasculitis, with the exception of aortitis and aneurysm or aortic insufficiency, occurring in about 12% of patients [8]. A definite diagnosis of CS is based upon characteristic involvement of both the eye and inner ear, supported by the histologic abnormalities and exclusion of other conditions.

We report a case of a typical Cogan's syndrome, the workup of the diagnosis, and treatment results.

## 2. Presentation Case

**Figure 1:**
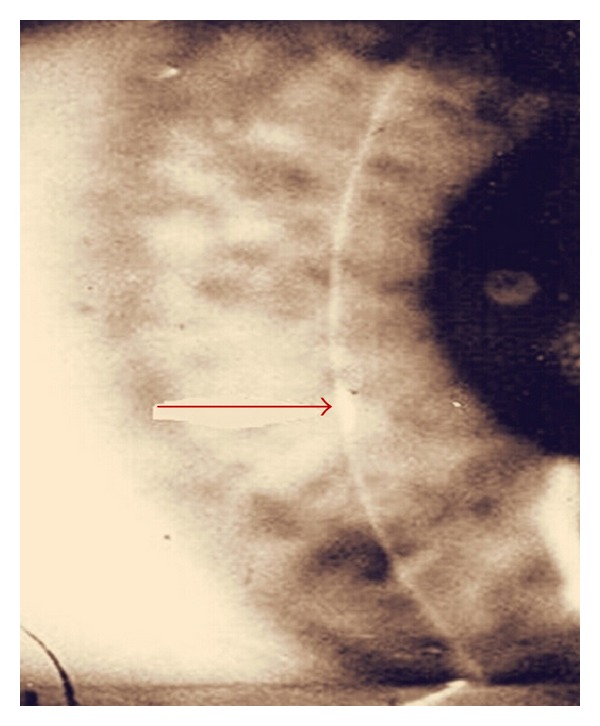
Slit lamp examination of the eye showed interstitial keratitis.

**Figure 2:**
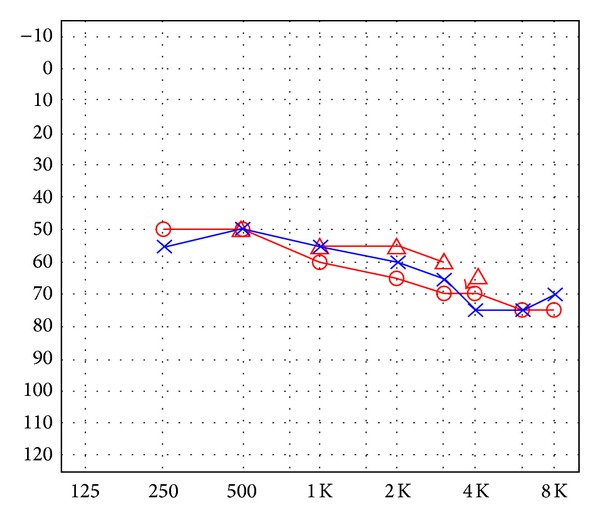
Audiogram showing bilateral moderate sensorineural hearing loss.

A 47-year-old female was hospitalized due to headache, vertigo, nausea, vomiting, right leg claudication, musculoskeletal pains, progressive bilateral hearing loss, and blindness for the past two months. At the time of admission, brachial blood pressure was 125/75 mmHg in the left arm and 120/70 mmHg in the right arm. The pulse rate was 85 beats per minute and respiration rate was 18 breaths per minute. Her body temperature was 37.1°C. Heart and lung sounds were normal. Ophthalmologic examination revealed that visual acuity was 0.1 bilaterally, and intraocular pressure was 15 mmHg in both eyes. On biomicroscopy, bilateral ciliary hyperemia, cataract, and interstitial keratitis with marginal infiltrates were noticed (Figure 1). The fundus examination revealed mild hyperemia of both optic discs, with blurred borders, and no signs of vitreous or retinal inflammation. Pure tone audiogram (PTA) and auditory brainstem responses (ABR) showed bilateral sensorineural hearing loss (Figure 2).

The laboratory test results were as follows: white blood cell count 10,200/mm^3^, hemoglobin 9.8 g/dL, platelet count 340,000/mm^3^, erythrocyte sedimentation rate 65 mm/hr, and C-reactive protein 13.1 mg/dL. She tested negative for autoimmune tests (anti-nuclear antibody, anti-neutrophil cytoplasmic antibody rheumatoid factors, anti-phospholipid antibodies, complements, and cryoglobulins) and negative for syphilis serology tests (VDRL, FTA-Abs). Laboratory tests and cerebrospinal fluid (CSF) analysis were all negative for an infection (antibodies IgG and IgM against Epstein-Barr virus, herpes zoster virus, herpes simplex virus, *Cytomegalovirus*, chlamydia, hepatitis viruses B and C, HIV, *Toxoplasma gondii*, and *Mycobacterium tuberculosis*). The cerebral magnetic resonance imaging (MRI), computer tomography of the chest and abdomen, colonoscopy, endoscopy, and biopsy of minor salivary glands were all normal.

Based on audiovestibular and ocular findings and given the excluding the infectious, neoplastic, granulomatous, and autoimmune etiologies, the diagnosis of “typical” Cogan's syndrome was established. After the treatment with intravenous methylprednisolone (1 gr/day for 5 days) and cyclophosphamide (1 gr/month for 7 months) her general condition stabilized, anterior eye segment changes improved, and inflammatory parameters remained in normal range. During the following six months, systemic prednisone was gradually reduced to 30 mg/d, cyclophosphamide was discontinued, and oral methotrexate 20 mg/week was given. However, clinical follow-up showed partial improvement.

## 3. Discussion

Cogan's syndrome (CS) was first described in 1945 by an ophthalmologist, Dr. Cogan, who reported on a syndrome of nonsyphilitic interstitial keratitis (IK) and vestibuloauditory symptoms that resembled Meniere's disease [1]. In addition to the ocular and audiovestibular involvement, numerous systemic manifestations were reported in 1960 by Cody and Williams in patients with CS [12]. More than 200 cases of CS have been reported in the literature, despite being a rare condition that mostly affects Caucasian young adults [11, 13].

In 1980, Haynes et al. [2] proposed diagnostic criteria for typical and atypical CS, which include a large spectrum of clinical manifestations. Typical CS is defined using Cogan's original criteria [1] with the following three conditions: (1) ocular symptoms, nonsyphilitic IK; (2) audiovestibular symptoms similar to those of Meniere's syndrome (sudden onset of tinnitus and vertigo, accompanied by gradual hearing loss); and (3) an interval between the onset of ocular and audiovestibular manifestations of less than 2 years. According to the criteria of Haynes et al. [2], patients with any of the following symptoms are classified as having atypical CS: (1) inflammatory ocular manifestations, with or without IK; (2) typical ocular manifestations associated with audiovestibular symptoms different from Meniere's-like episodes; or (3) a delay of more than 2 years between the onset of typical ocular and audiovestibular manifestations. In atypical CS, where the ocular manifestation is episcleritis, scleritis, iritis, uveitis, or chorioretinitis rather than interstitial keratitis, there is a worse prognosis and a higher frequency of aortic and other systemic manifestations [11].

Recurrent episodes of inner ear disease frequently result in profound hearing loss. In a retrospective series of 60 patients from one center, hearing loss was typically sudden, bilateral, fluctuating, and progressive, resulting in complete hearing loss in 73 of 120 ears [8]. Hearing loss in both ears was noted at some point in all patients. Two smaller studies both found bilateral deafness in approximately two-thirds of patients [2, 3]. Typically, audiometry testing demonstrates a sensorineural hearing loss, preferentially involving the low and high range frequencies; poor speech discrimination is also observed. In one preliminary study, at least 30 percent of patients had a pure tone audiometry threshold of greater than or equal to 60 dB, a threshold value indicative of moderately severe hearing loss [4]. Hearing loss is often bilateral from onset but in some patients it may be unilateral initially, becoming bilateral later. In the review by Vollertsen et al. [3], which included 78 patients with typical Cogan's syndrome, bilateral deafness affected 43.5% of patients and occurred a mean of 3 months after the onset of the initial symptoms.

When present, the systemic vasculitis associated with CS is a large- or medium- to small-sized vessel vasculitis, or an aortitis. The pattern of vessel involvement may be overlapping. Aortitis, which may develop within weeks to years of disease onset, has been described in approximately 10 percent of patients [2, 3, 11]. It may cause proximal aorta dilation, aortic valvular regurgitation, ostial coronary artery disease, and thoracoabdominal aortic aneurysms [11, 14–18]. A coronary arteritis has also been described [3, 19, 20]. The large vessel vasculitis associated with CS may also resemble Takayasu's arteritis, causing an occlusion of the aortic arch vessels with resultant upper and/or lower limb claudication, or renal artery stenosis [14, 19, 21, 22]. A small- or medium-sized vessel arteritis has been described in some cases [23].

Nonspecific systemic manifestations of CS include fever, fatigue, weight loss, lymphadenopathy, hepatomegaly, hepatitis, splenomegaly, pulmonary nodules, pericarditis, abdominal pain, arthralgia, arthritis, myalgia, and urticaria [4, 8, 10, 11]. The disorder has also been described in patients with inflammatory bowel disease [24, 25]. The differential diagnosis of CS includes diverse conditions that cause similar eye and inner ear manifestations (Table 1).

Therapeutic options for the treatment of CS include the use of topical agents for limited ocular disease and immunosuppressive therapy for more extensive ocular disease, inner ear involvement, and/or systemic vasculitis. Systemic corticosteroids are always the most widely used and successful therapy in Cogan's syndrome. For patients requiring high and prolonged doses, additional immunosuppression is appropriate. Methotrexate is the first-line steroid sparing agent [26, 27]. However, patients without systemic disease or severe eye disease unmanageable by topical corticosteroids should not be subjected to protracted courses of corticosteroids or immunosuppressive agents, particularly when little gain in hearing is obtained with their use [19]. The effect of TNF-alpha blockers was recently investigated. Infliximab might be an alternative therapy in cases of failure of corticosteroids and immunosuppressive therapy [28, 29]. However, treatment might be more effective when started at an early stage of the disease, when the lesions are still reversible. Surgical bypass grafting or aortic valve replacement may be required in some patients [20].

## 4. Conclusion

Our case report of CS demonstrates objective, simultaneous deterioration of hearing and vestibular function, which partially improved and stabilized after the introduction of immunosuppressive medication. The diagnosis of CS is largely based on clinical features, supported by the histologic abnormalities and exclusion of other conditions. Treatment might be more effective when started at an early stage of the disease.

## Figures and Tables

**Table 1:** Differential diagnosis of Cogan's syndrome.

Sarcoidosis
Congenital syphilis
Whipple's disease
Vogt-Koyanagi-Harada syndrome
KID (keratitis, ichthyosis, and deafness) syndrome
Sjögren's syndrome
Rheumatoid arthritis
Systemic lupus erythematosus
Granulomatosis with polyangiitis (Wegener's)
Polyarteritis nodosa
Ulcerative colitis, Crohn's disease
Central nervous system lymphoma/leukemia
Anti-phospholipid antibody syndrome
Behçet's syndrome
Chlamydial infection
Viral infection
Herpes simplex and varicella zoster infection
*Mycobacterium tuberculosis* infection
Demyelinating disease (e.g., multiple sclerosis)
Cerebellopontine angle tumor
